# Increase of *Plasmodium falciparum* parasites carrying lumefantrine-tolerance molecular markers and lack of South East Asian *pfk13* artemisinin-resistance mutations in samples collected from 2013 to 2016 in Côte d’Ivoire

**DOI:** 10.1007/s12639-023-01640-4

**Published:** 2024-01-12

**Authors:** Abibatou Konaté-Touré, Akpa Paterne Gnagne, Akoua Valérie Bedia-Tanoh, Eby Ignace Hervé Menan, William Yavo

**Affiliations:** 1 Department of Parasitology, Mycology, Animal Biology and, Zoology, Felix Houphouët-Boigny University, BPV 34, Abidjan, Côte d’Ivoire; 2 Malaria Research and Control Centre, National Institute of Public Health, BPV 47, Abidjan, Côte d’Ivoire

**Keywords:** Malaria, *Plasmodium falciparum*, Drug resistance, Molecular markers

## Abstract

One of the major obstacles to malaria elimination in the world is the resistance in *Plasmodium falciparum* to most antimalarial drugs. This study aimed to estimate the prevalence of molecular markers of antimalarial drugs resistance in Côte d’Ivoire. Samples were collected from 2013 to 2016 from asymptomatic and symptomatic subjects in Abengourou, Abidjan, Grand Bassam, and San Pedro. A total of 704 participants aged between 1 year and 65 years (Mean age: 9 years ± 7.7) were enrolled. All the dried filter paper blood spots were genotyped by sequencing. *Plasmodium falciparum kelch propeller domain 13 (pfk13*) gene were analyzed for all the samples, while 344 samples were examined for *Plasmodium falciparum multi-drug resistance 1 (pfmdr1)*. Overall, the success rate of molecular tests was 98.8% (340/344), 99.1% (341/344), and 94.3% (664/704) for *pfmdr*1 N86Y, *pfmdr*1 Y184F, and *pfk13* genes respectively. Molecular analysis revealed twenty (5.9%; 20/340) and 219 (64.2%; 219/341) mutant alleles for *pfmdr*1 86Y and *pfmdr*1 184 F, respectively. Twenty-nine mutations in *pfk13* gene (4.4%; 29/664) with 2.7% (18/664) of non-synonymous mutations was found. None of the mutations previously described in South East Asia (SEA) involved in *P. falciparum* resistance to artemisinin derivatives were observed in this study. According to year of collection, a decrease of the prevalence of *pfk13* mutation (from 3.6 to 1.8%) and *pfmdr1* N86Y mutation (from 8.5 to 4.5%) and an increase of mutant allele of *pfmdr1* Y184F proportion (from 39.8 to 66.4%) were found. Comparing to previous studies in the country, this study showed an increase in lumefantrine tolerance of *P. falciparum* strains. This demonstrates the importance of establishing a strong system for molecular surveillance of malaria in Côte d’Ivoire.

## Introduction

Malaria elimination remains threatened by the resistance of *P. falciparum* to most antimalarial drugs. To address this concern, most endemic countries have adopted artemisinin-based combination therapies (ACTs) as first-line treatment for uncomplicated malaria. Artesunate-amodiaquine (ASAQ) and artemether-lumefantrine (AL) were officially adopted as first-line treatment for uncomplicated malaria in Côte d’Ivoire since 2007 (Ministère de la Santé et de l’Hygiène Publique 2007). The efficacy of these combinations depends on the susceptibility of parasites to artemisinin derivatives and partners drugs. Artemisinin resistance has been confirmed in five countries in the Greater Mekong subregion, namely Cambodia, the Lao People’s Democratic Republic, Myanmar, Thailand, and Vietnam (Dondorp et al. 2011; Noedl et al. 2008; Plowe 2009). Recently, *P. falciparum* isolates with mutations in *Plasmodium falciparum kelch propeller domain 13 (pfk13)* gene linked to artemisinin resistance have been also reported in Rwanda and Tanzania (Uwimana et al. 2020; Bwire et al. 2020; Bergmann et al. 2021). Other sub-Saharan African countries including Côte d’Ivoire could similarly face the same phenomenon with a risk of increased malarial morbidity and mortality rates. The study of molecular markers is an early warning tool for drug resistance that can guide clinical efficacy studies on antimalarial drugs and malaria treatment guidelines (Ringwald et al. 2002). The molecular markers most commonly studied for antimalarial drug resistance are *Plasmodium falciparum chloroquine resistance transporter (pfcrt)*, *Plasmodium falciparum multi-drug resistance 1* (*pfmdr*1), and *pfk13*. *Pfcrt* is linked to chloroquine (CQ) and amodiaquine (AQ) resistance (Eyase et al. 2013). *Pfmdr*1 is involved in *P. falciparum* resistance to many antimalarial drugs, such as AQ and lumefantrine, which are used as partner drug for artemisinin derivatives in ASAQ and AL (Sisowath et al. 2005; Dokomajilar et al. 2006). *Pfk13* gene is a molecular marker of artemisinin resistance (WHO 2017). In Côte d’Ivoire, few recent data are available on the allelic frequencies of these genes. In the past, *pfcrt* 76T mutant allele accounted for 47.6–100% in Abidjan (the economic capital) (Djaman et al. 2004; Bla et al. 2014) and in its border towns (Ako et al. 2012; Ouattara et al. 2011). Our preliminary study carried on symptomatic subjects showed a prevalence of *pfcrt* gene mutation of 16.6% (Konaté et al. 2018a, 2018b). Three points of mutations (N86Y, Y184F, and D1246Y) of *pfmdr1* gene, with respectively frequencies ​​of 47.5%, 75%, and 1.6% were reported in Côte d’Ivoire in 2006–2007 [unpublished data]. None of the mutations in *pfk13* gene observed in South-East Asia (SEA) have been observed in Côte d’Ivoire (Kamau et al. 2014; Basco et al. 2017). Thus, this study aimed to update data by determining the prevalence of molecular markers of antimalarial drug resistance in Côte d’Ivoire.

## Materials and methods

### Study area

The study was conducted on samples collected at Abidjan, Grand-Bassam (coastal and forest zones), Abengourou (forest zone), and San Pedro (coastal and forest zones). Côte d’Ivoire experiences two wet seasons (from April to July and from October to November) and two dry seasons (from December to March and August to September). Malaria transmission in the country is perennial, with peaks occurring during wet seasons. According to the National Malaria Control Program (NMCP), malaria cases are predominantly caused by *P. falciparum* (95–99%), followed by *P. malariae* (3–4.2%) and *P. ovale* (0.3–0.7%) (Assi et al. 2013; Ministère de la Santé et de la Lutte contre le SIDA 2015).

### Study design

*Plasmodium falciparum* infected samples were collected from asymptomatic and symptomatic individuals from 2013 to 2016. Molecular analysis was performed from May 2016 to November 2018 at the Malaria Research and Control Center located at the National Institute of Public Health (Abidjan, Côte d’Ivoire).

Samples from symptomatic subjects were collected through routine evaluation of antimalarial drugs at public health facilities in Abidjan (2013), and Abengourou and San Pedro (2016). The inclusion criteria of symptomatic patients were: an age between 6 months to 65 years, with uncomplicated malaria and a monospecific *P. falciparum* infection diagnosed by microscopy. Another requirement was that the patients must have maintained residence in the study area for at least 2 months.

Asymptomatic subjects were recruited at primary school in Abengourou, Grand-Bassam and San Pedro (2015–2016). The group largely comprised school children aged between 4 and 16 years. Any non-febrile (temporal temperature below 37.8 ºC) subject carrying the asexual form of *Plasmodium* without any clinical signs of malaria during the seven days before and after medical examination was considered asymptomatic.

### Malaria confirmation

To confirm *Plasmodium* carriage and determine parasitemia, thick and thin blood films were performed. The density of *P. falciparum* in peripheral blood was determined by counting the number of asexual parasites for 200 white blood cells per µL; i.e., number of parasites × 8000/200 assumed a white blood cell mean of 8,000 cells per µL as recommended by WHO when the patient’s exact white blood cell count is not available (WHO 2010). A double-check reading was performed for all slides.

### Dried filter paper blood spots collection

For molecular analysis and prior to any treatment (symptomatic subjects), approximately 50 µL of whole blood was collected from the subjects’ fingertips and blotted on sterile filter paper (N°3; Whatman International Ltd. Maidstone, UK). The blotted filter paper was then dried secluded from insects and dust for at least 24 h. Dried blood spots were then stored at room temperature, protected with a silica-gel desiccant, and archived for molecular analysis.

### DNA extraction

Parasite DNA was extracted from dried blood spots using the QIAamp® DNA Blood Mini Kit (Qiagen, Hilden, Germany) and the Quick-DNA™ Universal Kit (Zymo Research, California, USA) according to the manufacturer’s instructions.

### Genotyping of ***pfmdr***1 and ***pfk13 ***genes

*Pfk13* gene was analyzed on all the dried blood spots, while *pfmdr1* polymorphism was studied on dried blood spots from Abidjan, Abengourou, and San Pedro. Nested PCR was done for all the samples. The manufacturer’s instructions were followed throughout the process. The first mix contained for each amplification 12.5 µL of the master mix named OneTaq® 2X Master Mix with Standard Buffer (New England Biolabs, Ipswich, MA, USA), 0.5 µL of each primer (10 µM), 6.5 µL of Nuclease free water, and 5 µL of DNA template. The second amplification used a final volume of 50 µL final volume sufficient for sequencing. The primer sequences are listed in Table 1. After the second amplification, electrophoresis enabled visualization of PCR products on a 1.5% agarose gel. Electrophoresis was performed at 100 mV for 45 min. All *pfmdr*1 and *pfk13* PCR products that were successfully amplified and then verified by the visualization of a fragment after migration were selected for Sanger sequencing.

**Table 1:** Primers sequences of *pfmdr1* and *pfk13 propeller* genes

Primers	Sequences	Genes
MDR1_PCR1_F	5′-AGAGAAAAAAGATGGTAACCTCAG- 3′	*pfmdr*1
MDR1_PCR1_R	5′-ACC-ACA-AAC-ATA-AAT-TAA-CGG- 3′	*pfmdr*1
MDR1_PCR2_F	5′-TTTGTATGTGCTGTATTATCAGG- 3′	*pfmdr*1
MDR1_PCR2_R	5′-GTA-ATA-CAT-AAA-GTC-AAA-CGT-GC- 3′	*pfmdr*1
K13_PCR_F	5′- CGGAGTGACCAAATCTGGGA − 3′	*pfk13*
K13_PCR_R	5′- GGGAATCTGGTGGTAACAGC − 3′	*pfk13*
K13_N1_F	5′- GCCAAGCTGCCATTCATTTG − 3′	*pfk13*
K13_N1_R	5′- GCCTTGTTGAAAGAAGCAGA − 3′	*pfk13*

### Sequencing and sequences analysis

The PCR products were sequenced according to the Sanger sequencing method by the company Genewiz (Takeley, United Kingdom). The sequences were analyzed by alignment with the BioEdit™ software using pf3D7_1343700 (NCBI) as a reference strain (Reference sequence accession number: XM_001350122 for *pfk13* and S53996 for *pfmdr1*). Codons 86 and 184 for the *pfmdr1* gene were analyzed while the entire sequence was analyzed for *pfk13* gene.

### Ethical considerations

The study was approved by the National Research Ethics Committee (Certificates numbers: N° 020/MSLS/CNER-dkn, N˚ 049/MSLS/CNER-dkn). The study was conducted in accordance with the principles of the Helsinki Declaration adopted by the 18th World Medical Assembly in 1964, its amendments (2000, amended in Tokyo in 2004), and the International Conference on Harmonization (ICH) recommendations. The design and execution of our study has been Good Clinical Practice compliant and all applicable regulatory requirements for clinical studies, as well as Côte d’Ivoire’s national laws and regulations have been met. Free and written informed consent was obtained from patients, parents, or legal guardians prior to the participant’s enrolment.

### Statistical analysis

SPSS Statistics version 21 (IBM Corp., Armonk, NY, USA) was used for data analysis. Different parameters were compared using the Fisher’s exact test. The level of significance for the statistical tests was set at 0.05. The polymorphism of *pfmdr1* and *pfk13* genes was determined based on the presence or absence of the wild-type and mutant alleles at the loci.

## Results

**Fig. 1:**
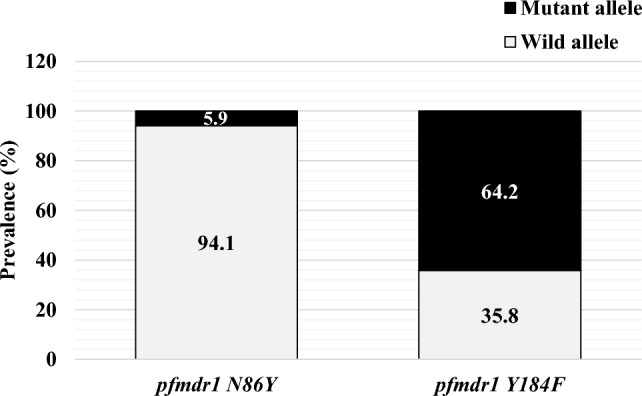
Allele frequencies of *pfmdr1* gene

A total of 704 participants were enrolled in this study. The study population comprised 460 symptomatic subjects and 244 asymptomatic subjects, with a mean age of 9 years (SD = 7.7). The minimum and maximum ages recorded were 1 year and 65 years, respectively. The geometric mean parasite count was 4,953 (SD = 43,066; range, 16–240,000) trophozoites per microliter of blood. All samples were analyzed for *pfk13* while 344 (all from symptomatic patients) were examined for *pfmdr*1. Overall, the success rates of molecular tests for *pfmdr*1 N86Y, *pfmdr*1 Y184F, and *pfk13* were 98.8% (340/344), 99.1% (341/344), and 94.3% (664/704), respectively. Twenty (5.9%) 86Y and 219 (64.2%) 184 F mutant alleles of the *pfmdr1* gene were found (Fig. 1). In addition, 29 mutants (4.4%; 29/664) of *pfk13* gene were found, with 18 non-synonymous mutations (2.7%).

According to year of collection (from 2013 to 2016), a decrease of the prevalence of *pfk13* mutation (from 3.6 to 1.8%) and *pfmdr1* N86Y mutation (from 8.5 to 4.5%) was noticed while an increase of mutant allele of *pfmdr1* Y184F proportion (from 39.8 to 66.4%) was found. The prevalence of *pfk13* gene mutations was significantly associated with parasite density (*p* = 0.003; Table 2). A578S (three cases) and G665S (two cases) were the most frequently detected mutations. In asymptomatic participants, the prevalence of *pfk13* gene mutations was 2.1% (5/243), of which 0.8% (2/243) were non-synonymous (Table 3). In the symptomatic group, the prevalence of *pfk13* gene mutations was 5.7% (24/421), of which 2.1% (9/421) were non-synonymous. For *Pfmdr1* gene, five non-common points of mutations were found apart from *Pfmdr1 N86Y* and *Pfmdr1 Y184F*: four cases of *Pfmdr1 R133K* and one (1) case of *Pfmdr1 D156Y.*

**Table 2:** Prevalence of *pfk13* and *pfmdr1* polymorphisms according to age and parasitemia

	*pfk13* non synonymous mutant type (n, %)	*p-value	*pfmdr*1 86Y (n, %)	*p-value	*pfmdr*1 184 F (n, %)	*p-value
Age (year)						
≤ 5	4 (2.3)		10 (5.7)		111 (63.4)	
6–14	5 (1.2)	*0.44*	5 (4.8)	*0.69*	65 (62.5)	*0.64*
≥ 15	2 (2.9)		5 (8.1)		43 (69.4)	
Sex						
Female	4 (1.2)	*0.37*	9 (5.7)	*0.64*	113 (63.8)	*0.91*
Male	7 (2.2)		11 (5.1)		106 (64.6)	
Parasitemia (parasites/µL)						
< 2,000	2 (0.5)		0 (0)		5 (71.4)	
2001–10,000	1 (1.3)		6 (6.3)		70 (73.7)	
10,001–25,000	1 (1.5)	***0.003***	6 (8.2)	*0.43*	49 (67.1)	*0.09*
25,001–50,000	4 (7.7)		5 (8.6)		33 (56.9)	
> 50,000	3 (3)		3 (2.8)		62 (57)	
Clinical status						
Symptomatic	9 (2.1)	*0.34*	–	–	–	–
Asymptomatic	2 (0.8)		–		–	
Location						
Abidjan	3 (1.8)		10 (8.5)		71 (60.2)	
Abengourou	4 (1.5)	*0.51*	5 (4.8)	*0.33*	70 (66.7)	*0.52*
Grand-Bassam	0 (0)		–		–	
San Pedro	4 (1.6)		5 (4.2)		78 (66.1)	
Date of collection						
2013	4 (3.6)		10 (8.5)		71 (39.8)	
2015	0 (0)	*0.13*	–	*0.15*	–	***0.002***
2016	8 (1.8)		10 (4.5)		148 (66.4)	
**Drug treatment						
ASAQ	5 (3.2)	*1.00*	14 (8.3)	*0.10*	105 (61.4)	*0.31*
AL	4 (2.7)		6 (3.6)		114 (67.1)	

The bold values represent the results statistically significant

*Fisher’s exact test.

**only for symptomatic subjects

**Table 3:** Non synonymous mutations in *pfk13* gene

Codon position	Amino acid reference sequence	Nucleotide reference sequence	Amino acid mutant-type sequence	*Nucleotide mutant-type sequence	Prevalence n (%)
475	P	CCT	H	C**A**T	1 (0.2)
482	Y	TAT	H	**C**AT	1 (0.2)
522	S	AGT	C	**T**GT	1 (0.2)
538	G	GGT	S	**A**GT	1 (0.2)
557	A	GCA	S	**T**CA	1 (0.2)
578	A	GCG	S	**T**CG	3 (0.5)
665	G	GGC	S	**A**GC	2 (0.3)
676	A	GCC	S	**T**CC	1 (0.2)

*In bold: Single nucleotide polymorphism in pfK13 gene

## Discussion

Regular monitoring of *P. falciparum* resistance molecular markers is a key factor in the early detection of emerging resistance and achieving malaria elimination. In this study, *pfmdr*1 and *pfk13* genes were studied for their implication in *P. falciparum* resistance to antimalarial drugs, particularly amodiaquine, lumefantrine, and artemisinin derivatives. The limitation of this study was the lack of data to investigate the temporal comparison in the same site to identify subnational trends in prevalence in different geographic areas.

A selection for *pfmdr1* 86 N wild-type alleles was observed as a result of using ASAQ and AL for many years. This selection is commonly associated with lumefantrine tolerance (Raman et al. 2019). Indeed, *pfmdr*1 polymorphism is usually studied because of its involvement in *P. falciparum*’s resistance to many antimalarial drugs, particularly those used as partner drugs of artemisinin derivatives in ASAQ and AL (amodiaquine and lumefantrine). Single-nucleotide polymorphisms (SNPs) at codons 86 (N → Y), 184 (Y → F), and 1246 (D → Y) of *pfmdr1* are among the most observed. Monitoring of NFD and YYY haplotypes is useful for detecting an early decrease in susceptibility to lumefantrine and amodiaquine, respectively (Venkatesan et al. 2014). Usually, after the change from chloroquine to ACTs, an increase in parasites carrying *pfmdr1* 86 N was found, as reported in this study from 2013 to 2016 (Okell et al. 2018). In the same way, a previous study conducted in 2006 i.e. one year before the official adoption of ACTs as first-line treatment for uncomplicated malaria cases showed a higher value of the *mdr1*86Y allele (47.5%) than ours (unpublished data). In addition, an upward trend of *mdr*184F mutant-allele prevalence is usually reported after the adoption of ACTs, as observed in the present study from 2013 to 2016 (Okell et al. 2018). The prevalence of this mutation in Abidjan in 2010 was 57% (Trebissou et al. 2014), which was lower than ours. In this study, we did not investigate the *mdr*D1246Y mutation. A previous study carried out in Côte d’Ivoire on this mutation reported a low incidence of mutant (1.6%) [unpublished data]. A scarcity of mutant *mdr*1246Y has also been found in Burkina Faso (Sondo et al. 2016). These results point to lumefantrine tolerance in strains circulating through the country, as found in many reports from sub-Saharan African areas that use AL combination (Raman et al. 2019; Sondo et al. 2016; Dama et al. 2017). The *pfmdr*1 gene results observed in this study could be due to frequent use of AL combination over AS + AQ due to patient complaints following the use of the latter (Azagoh-Kouadio et al. 2017). Moreover, ex vivo susceptibility tests of *P. falciparum* strains circulating in Côte d’Ivoire should confirm this result. In Mali, a reduced ex vivo susceptibility of *P. falciparum* was observed after AL treatment (Dama et al. 2017).

Mutations in *pfk13* gene (C580Y, R561H, R539T, I543T, Y493H, M476I, N458Y, Y493H, and recently F446I) (Wang et al. 2018) are molecular markers of artemisinin resistance validated by in vitro and in vivo studies, while P441L, G449A, G538V, P553L, R561H, V568G, P574L, A578S, and A675V are candidate markers (WHO 2017). These validated mutations have been reported in Cambodia and other SEA countries and serve as tools for monitoring ACTs resistance (Ariey et al. 2014). These mutations are absent in this study as well as in a previous study conducted in 2017 in Abidjan (Basco et al. 2017). This result suggests that *P. falciparum* parasites circulating in Côte d’Ivoire are still sensitive to artemisinin. This is consistent with the findings of previous results from clinical trials at sentinel sites in the country that have shown adequate clinical and parasitological responses of up to 100% (Toure et al. 2014; Yavo et al. 2015; Konaté et al. 2018a). However, recent studies in Rwanda and Tanzania have showed the presence of R561H mutations (Uwimana et al. 2020; Bwire et al. 2020; Bergmann et al. 2021). In addition, phylogenetic analysis of Rwanda strains revealed the expansion of an indigenous R561H lineage and evidence for the *de novo* emergence of Pfkelch13-mediated artemisinin resistance in Rwanda (Uwimana et al. 2020, 2021). These data highlight the importance of surveying antimalarial molecular markers in Côte d’Ivoire. Additional mutations were also identified in this study. Indeed, A557S, a non-synonymous mutation already reported in Côte d’Ivoire (Kamau et al. 2014) and Congo by Taylor et al. (Taylor et al. 2015), was found in this study. In addition, the S522C mutation observed in this study has already been described in Africa, particularly West Africa (Togo), Central Africa (Central African Republic, Gabon, Democratic Republic of Congo), and East Africa (Kenya) (Menard et al. 2016). The A578S mutation, a candidate marker for artemisinin resistance (WHO 2017), has the highest prevalence in this study (0.5%). This mutation seems to be the most widespread worldwide. Indeed, it has been reported in a small proportion in Cambodia (Straimer et al. 2015) and several sub-Saharan African countries, including the Democratic Republic of Congo (Kamau et al. 2014; Mayengue et al. 2018), Uganda (Conrad et al. 2014), Equatorial Guinea (Li et al. 2016), Gabon (Kamau et al. 2014), Ghana (Matrevi et al. 2019), Kenya (Kamau et al. 2014), Togo (Dorkenoo et al. 2016), and Mali (Kamau et al. 2014; Dama et al. 2017). In vitro studies have confirmed that this mutation is not responsible for artemisinin resistance (Straimer et al. 2015; de Laurent et al. 2018; Ashley et al. 2014). However, because of its common occurrence in many countries, further characterization, as well as an assessment of its role in in vivo parasite clearance in sub-Saharan African countries is required. Moreover, the mutations P475H, Y482H, G665S, and A676S reported in our study have not been observed before. These results indicate that in Africa, parasites show a high polymorphism in *pfk13* gene; however, the mutations observed are different from those reported in SEA. These differences seem to be affected by demographic and epidemiological parameters (Joy et al. 2003) rather than differential selective pressures that may be transient, and not necessarily due to artemisinin pressure (de Laurent et al. 2018). Although some candidate marker mutations have been reported in Africa, they were not involved in any case of treatment failure. Thus, the molecular mechanisms underlying artemisinin resistance remain unclear (Takala-Harrison et al. 2013). This phenomenon is likely to involve multiple genetic loci (Ariey et al. 2014; Miotto et al. 2015), and requires complete genotyping of circulating *P. falciparum* strains to detect new markers. Further studies, such as site-specific genome editing experiments using zinc finger nucleases (Mayengue et al. 2018), or the CRISPR-Cas9 system (Ghorbal et al. 2014), could shed light on the role of mutations discovered in sub-Saharan Africa, particularly in Côte d’Ivoire, in the resistance of *P. falciparum* to artemisinin derivatives.

## Conclusion

This study showed that the adoption of ACTs as first-line treatment for uncomplicated malaria cases results in a significant change in the allele frequencies of molecular markers responsible for *P. falciparum* resistance against ASAQ and AL. For *pfk13* gene, the mutations described in SEA and those associated with the resistance of *P. falciparum* to artemisinin derivatives were not observed in this study. An increase in the number of strains carrying the wild-type *pfmdr1* N86 allele indicated lumefantrine tolerance. These results could throw the efficacy of artemether–lumefantrine into question, and thus demonstrate the importance of establishing a strong system for malarial molecular surveillance in Côte d’Ivoire.

## References

[CR1] Ako BA, Offianan AT, Johansson M (2012). Molecular analysis of markers associated with chloroquine and sulfadoxine/pyrimethamine resistance in *Plasmodium falciparum* Malaria parasites from southeastern Cote d’Ivoire by the time of artemisinin-based combination therapy adoption in 2005. Infect Drug Resist.

[CR2] Ariey F, Witkowski B, Amaratunga C (2014). A molecular marker of artemisinin-resistant *Plasmodium falciparum* Malaria. Nature.

[CR3] Ashley EA, Dhorda M, Fairhurst RM (2014). Spread of artemisinin resistance in *Plasmodium falciparum* Malaria. N Engl J Med.

[CR4] Assi S-B, Henry M-C, Rogier C (2013). Inland Valley rice production systems and Malaria infection and disease in the forest region of western Côte d’Ivoire. Malar J.

[CR5] Azagoh-Kouadio R, Enoh S, Kassi K (2017). Paludisme De L’enfant: prise en charge Au Chu De Treichville. Rev Int Sci Médicales.

[CR6] Basco LK, Tahar R, Ako AB (2017). Molecular epidemiology of Malaria in Cameroon and Côte d’Ivoire. XXXI. Kelch 13 propeller sequences in *Plasmodium falciparum* isolates before and after implementation of artemisinin-based combination therapy. Am J Trop Med Hyg.

[CR7] Bergmann C, van Loon W, Habarugira F (2021). Increase in Kelch 13 polymorphisms in *Plasmodium falciparum*, Southern Rwanda. Emerg Infect Dis.

[CR8] Bla BK, Yavo W, Trébissou J (2014). Polymorphisms of the pfatpase 6 and pfcrt gene and their relationship with the in vitro susceptibility to dihydroartemisinin and chloroquine of *Plasmodium falciparum* isolates from Abobo, Côte d’Ivoire. Ann Parasitol.

[CR9] Bwire GM, Ngasala B, Mikomangwa WP (2020). Detection of mutations associated with artemisinin resistance at k13-propeller gene and a near complete return of chloroquine susceptible falciparum Malaria in Southeast of Tanzania. Sci Rep.

[CR10] Conrad MD, Bigira V, Kapisi J (2014). Polymorphisms in K13 and Falcipain-2 Associated with Artemisinin resistance are not prevalent in *Plasmodium falciparum* isolated from Ugandan Children. PLoS ONE.

[CR11] Dama S, Niangaly H, Ouattara A (2017). Reduced ex vivo susceptibility of* Plasmodium falciparum* after oral artemether–lumefantrine treatment in Mali. Malar J.

[CR150] Dondorp AM, Fairhurst RM, Slutsker L (2011). The threat of artemisinin-resistant malaria. N Engl J Med.

[CR12] Djaman J, Abouanou S, Basco L, Koné M (2004). Limits of the efficacy of chloroquine and sulfadoxine-pyrimethamine in Northern Abidjan (Cote d’Ivoire): combined in vivo and in vitro studies. Sante Montrouge Fr.

[CR13] Dokomajilar C, Nsobya SL, Greenhouse B (2006). Selection of *Plasmodium falciparum* pfmdr1 alleles following therapy with artemether-lumefantrine in an area of Uganda where Malaria is highly endemic. Antimicrob Agents Chemother.

[CR14] Dorkenoo AM, Yehadji D, Agbo YM (2016). Therapeutic efficacy trial of artemisinin-based combination therapy for the treatment of uncomplicated Malaria and investigation of mutations in k13 propeller domain in Togo, 2012–2013. Malar J.

[CR15] Eyase FL, Akala HM, Ingasia L (2013). The role of Pfmdr1 and pfcrt in changing chloroquine, amodiaquine, mefloquine and lumefantrine susceptibility in Western-Kenya P. Falciparum samples during 2008–2011. PLoS ONE.

[CR16] Ghorbal M, Gorman M, Macpherson CR (2014). Genome editing in the human Malaria parasite *Plasmodium falciparum* using the CRISPR-Cas9 system. Nat Biotechnol.

[CR17] Joy DA, Feng X, Mu J (2003). Early origin and recent expansion of lasmodium falciparum. Science.

[CR18] Kamau E, Campino S, Amenga-Etego L (2014). K13-Propeller polymorphisms in *Plasmodium falciparum* parasites from Sub-saharan Africa. J Infect Dis.

[CR19] Konaté A, Barro-Kiki PCM, Angora KE (2018). Efficacy and tolerability of artesunate-amodiaquine versus artemether-lumefantrine in the treatment of uncomplicated *Plasmodium falciparum* Malaria at two sentinel sites across Côte d’Ivoire. Ann Parasitol.

[CR20] Konaté A, Gnagne PA, Bédia-Tanoh VA (2018). Low rates of *Plasmodium falciparum* Pfcrt K76T mutation in three sentinel sites of Malaria monitoring in Côte d’Ivoire. Acta Parasitol.

[CR21] de Laurent ZR, Chebon LJ, Ingasia LA (2018). Polymorphisms in the K13 gene in *Plasmodium falciparum* from different Malaria transmission areas of Kenya. Am J Trop Med Hyg.

[CR22] Li J, Chen J, Xie D (2016). Limited artemisinin resistance-associated polymorphisms in *Plasmodium falciparum* K13-propeller and PfATPase6 gene isolated from Bioko Island, Equatorial Guinea. Int J Parasitol Drugs Drug Resist.

[CR23] Matrevi SA, Opoku-Agyeman P, Quashie NB (2019). Plasmodium Falciparum Kelch Propeller Polymorphisms in clinical isolates from Ghana from 2007 to 2016. Antimicrob Agents Chemother.

[CR24] Mayengue PI, Niama RF, Kouhounina Batsimba D (2018). No polymorphisms in K13-propeller gene associated with artemisinin resistance in *Plasmodium falciparum* isolated from Brazzaville, Republic of Congo. BMC Infect Dis.

[CR25] Menard S, Tchoufack JN, Maffo CN (2016). Insight into k13-propeller gene polymorphism and ex vivo DHA-response profiles from Cameroonian isolates. Malar J.

[CR26] Ministère de la Santé et de l’Hygiène Publique (2007). Arrêté n°24/CAB/MSHP Du 12 janvier 2007 portant institution d’un schéma thérapeutique pour traitement du paludisme en Côte d’Ivoire.

[CR27] Ministère de la Santé et de la Lutte contre le SIDA (2015). Plan stratégique national de lutte contre le paludisme 2012–2015 révisé (période replanifiée): Approche stratifiée De mise à échelle des interventions de lutte contre le paludisme en Côte d’Ivoire et consolidation des acquis.

[CR28] Miotto O, Amato R, Ashley EA (2015). Genetic architecture of artemisinin-resistant *Plasmodium falciparum*. Nat Genet.

[CR200] Noedl H, Se Y, Schaecher K (2008). Evidence of artemisinin-resistant malaria in western Cambodia. N Engl J Med.

[CR29] Okell LC, Reiter LM, Ebbe LS (2018). Emerging implications of policies on Malaria treatment: genetic changes in the Pfmdr-1 gene affecting susceptibility to artemether–lumefantrine and artesunate–amodiaquine in Africa. BMJ Glob Health.

[CR30] Ouattara L, Bla K, Assi S (2011). PFCRT and DHFR-TS sequences for monitoring drug resistance in Adzopé area of Côte d’Ivoire after the withdrawal of Chloroquine and Pyrimethamine. Trop J Pharm Res.

[CR250] Plowe CV (2009). The evolution of drug-resistant malaria. Trans R Soc Trop Med Hyg.

[CR31] Raman J, Kagoro FM, Mabuza A (2019). Absence of kelch13 artemisinin resistance markers but strong selection for lumefantrine-tolerance molecular markers following 18 years of artemisinin-based combination therapy use in Mpumalanga Province, South Africa (2001–2018). Malar J.

[CR32] Ringwald P, Sukwa T, Basco LK (2002). Monitoring of drug-resistant Malaria in Africa. Lancet.

[CR33] Sisowath C, Strömberg J, Mårtensson A (2005). In vivo selection of *Plasmodium falciparum pfmdr1* 86 N coding alleles by artemether-lumefantrine (Coartem). J Infect Dis.

[CR34] Sondo P, Derra K, Diallo Nakanabo S (2016). Artesunate-amodiaquine and artemether-lumefantrine therapies and selection of Pfcrt and Pfmdr1 alleles in Nanoro, Burkina Faso. PLoS ONE.

[CR35] Straimer J, Gnädig NF, Witkowski B (2015). K13-propeller mutations confer artemisinin resistance in *Plasmodium falciparum* clinical isolates. Science.

[CR36] Takala-Harrison S, Clark TG, Jacob CG (2013). Genetic loci associated with delayed clearance of *Plasmodium falciparum* following artemisinin treatment in Southeast Asia. Proc Natl Acad Sci U S A.

[CR37] Taylor SM, Parobek CM, DeConti DK (2015). Absence of putative artemisinin resistance mutations among *Plasmodium falciparum* in Sub-saharan Africa: a molecular epidemiologic study. J Infect Dis.

[CR38] Toure OA, Assi SB, N’Guessan TL (2014). Open-label, randomized, non-inferiority clinical trial of artesunate-amodiaquine versus artemether-lumefantrine fixed-dose combinations in children and adults with uncomplicated falciparum Malaria in Côte d’Ivoire. Malar J.

[CR39] Trebissou JND, Yavo W, Tano KD (2014). In vitro susceptibility of *Plasmodium falciparum* to lumefantrine and analysis of polymophysims in *pfmdr-1* gene isolates from Abidjan (Côte d’Ivoire). Int J Pharm Sci Res.

[CR40] Uwimana A, Legrand E, Stokes BH (2020). Emergence and clonal expansion of in vitro artemisinin-resistant *Plasmodium falciparum* kelch13 R561H mutant parasites in Rwanda. Nat Med.

[CR41] Uwimana A, Umulisa N, Venkatesan M (2021). Association of *Plasmodium falciparum* kelch13 R561H genotypes with delayed parasite clearance in Rwanda: an open-label, single-arm, multicentre, therapeutic efficacy study. Lancet Infect Dis.

[CR42] Venkatesan M, Gadalla NB, Stepniewska K (2014). Polymorphisms in *Plasmodium falciparum* chloroquine resistance transporter and multidrug resistance 1 genes: parasite risk factors that affect treatment outcomes for P. Falciparum Malaria after artemether-lumefantrine and artesunate-amodiaquine. Am J Trop Med Hyg.

[CR43] Wang J, Huang Y, Zhao Y (2018). Introduction of F446I mutation in the K13 propeller gene leads to increased ring survival rates in *Plasmodium falciparum* isolates. Malar J.

[CR44] WHO (2010). Basic malaria microscopy – Part I: Learner’s guide.

[CR45] WHO (2017) World Malaria report 2017. Geneva

[CR46] Yavo W, Konaté A, Kassi FK (2015). Efficacy and safety of Artesunate-Amodiaquine versus Artemether-Lumefantrine in the treatment of uncomplicated *Plasmodium falciparum* malaria in sentinel sites across Côte d’Ivoire. Malar Res Treat.

